# Treatment of Typhoid Fever by Carbolic Acid and Chloroform

**Published:** 1894-07

**Authors:** 


TREATMENT OF TYPHOID FEVER BY CARBOLIC ACID AND CHLOROFORM.—Dr. R. Quill (*Sem. Med.*, 1894; XIV.; p. 110). The author has, in treating typhoid fever, resorted to the simultaneous use of carbolic acid and chloroform, each of which, employed by itself, has already yielded good results in dothinenteritis. He employs the following formula:

| | | |
|---|---|---|
| Carbolic acid (pure) | 2.4 gme. | (37 grn.). |
| Spirit chloroform | 8 " | (2½ fl. drs.). |
| Tincture cardamom | 12 " | (3 fl. drs). |
| Syrup | 60 " | (1½ fl. oz.). |
| Saturated chloroform-water, | 275 " | (9 fl. oz.). |

Two tablespoonfuls in a little ice-water every two hours.

The patient takes, on the first day, two tablespoonfuls of this potion five times in the course of twenty-four hours; he repeats the same dose seven times on the next day, and ten times from the third day on, until a marked fall of temperature with a corresponding improvement of the general condition has set in. This result having once been obtained, the daily dose is gradually diminished to six tablespoonfuls and continued for at least a week after the temperature has become normal.

This mode of treatment is said to shorten the duration of the affection, to remove complications, and to rapidly suppress intestinal tympanites, diarrhea, fetor of the stools, delirium, and coma; also to promote the assimilation of food and to hasten convalescene.

Carbolic acid, as administered in the above-mentioned doses, is said to have always been well tolerated; and the urine hardly ever became darker, and never assumed a blackish color.—*Ex.*

